# Clinico-Serological Profile of Infective Causes of Acute Hepatitis in Children Admitted to a Tertiary Care Centre

**DOI:** 10.7759/cureus.38237

**Published:** 2023-04-28

**Authors:** Harsh Bal Gupta, Trupti Deshpande, Nirmal Choraria, Putun Patel, Shruti G Sethia, Soumitra Sethia

**Affiliations:** 1 Department of Paediatric Medicine, Cloud Nine Hospital, Panchkhula, IND; 2 Department of Paediatric Medicine, Gujarat Medical Education and Research Society (GMERS) Gotri, Vadodara, IND; 3 Department of Paediatric Medicine, Nirmal Hospital Pvt. Ltd., Surat, IND; 4 Department of Paediatric Medicine, Nandkumar Singh Chouhan Government Medical College, Khandwa, IND; 5 Department of Community Medicine, Nandkumar Singh Chouhan Government Medical College, Khandwa, IND

**Keywords:** hepatic encephalopathy, hepatitis e, hepatitis a, acute infective hepatitis, acute viral hepatitis

## Abstract

Background: Hepatitis is a major cause of healthcare burden in India. Hepatitis A is the most common cause of acute viral hepatitis in the pediatric population whereas hepatitis E virus (HEV) is the most important cause of epidemic hepatitis. Various other causes of acute infective hepatitis in children are dengue, malaria, and enteric fever. The aim of the present study is to understand the clinico-serological profile in cases of acute infective hepatitis in children.

Methodology: The present study is a cross-sectional study that was carried out from 1 September 2017 to 31 March 2019. A total of 89 children in the age group 1-18 years with clinically suspected acute infective hepatitis and subsequent confirmation on laboratory tests were included in the study.

Results: Hepatitis A (48.3%) was found to be the most common aetiology followed by dengue (22.5%) and hepatitis E (12.4%). No cases of hepatitis B or hepatitis C were found. The most common presenting complaint was fever (90%) and the most common clinical finding was icterus (69.7%). The sensitivity of icterus for the diagnosis of hepatitis was found to be 70%. Lab investigations showed a significant association between different etiologies of infective hepatitis with packed cell volume (PCV), white blood cell (WBC) count, and platelet count. Levels of aspartate aminotransferase (AST) and alanine transaminase (ALT) were raised in samples of patients with hepatitis A, hepatitis E, and combined hepatitis A and E infection as compared to other causes. All cases of hepatitis A and E were diagnosed with positive IgM antibody tests to the respective viral antigens. The most common complication was hepatic encephalopathy which was seen in patients with hepatitis A, dengue, and septicemia. Around 99% of patients recovered well and were discharged. One death occurred in a case of septicemia with septic shock with multiple organ dysfunction syndrome (MODS).

Conclusion: The most common cause of infective hepatitis in children is hepatitis A. Other causes like dengue, malaria, and typhoid should also be kept in mind. The absence of icterus does not rule out hepatitis. Lab investigations including serology are important to confirm the diagnosis of various causes of hepatitis. Timely immunization against hepatitis is strongly recommended.

## Introduction

Hepatitis is a term for inflammatory diseases of the liver, grossly subdivided into infectious and noninfectious, which are characterized by a wide variety of clinical and histological manifestations, ranging from mild and self-limited, to severe and progressive forms leading to liver failure, cirrhosis, or hepatocellular carcinoma [1].

Many viruses and agents (bacteria: Neisseria meningitidis, Neisseria gonorrhoeae, Salmonella species; fungi: Candida spp.; protozoans: Trypanosoma cruzi, Plasmodium species) can cause hepatitis. These include herpes simplex virus (HSV), cytomegalovirus, Epstein-Barr virus, varicella-zoster virus, human immunodeficiency virus (HIV), rubella, adenoviruses, enteroviruses, parvovirus B19, and arboviruses. Noninfectious causes of hepatitis include autoimmune causes (like autoimmune hepatitis), metabolic causes (like Wilson disease, tyrosinemia), drug-induced hepatitis, biliary atresia, etc [2]. Among all causes, viral hepatitis is a widespread cause of hepatitis. Hepatitis B and C if lasting more than six months are considered chronic whereas it is three months for hepatitis E. Chronic hepatitis can eventually progress to liver cirrhosis, liver failure, and hepatocellular carcinoma [1]. Viral hepatitis is a cause for major healthcare burden in India and is now equated as a threat comparable to the “big three” communicable diseases - HIV/AIDS, malaria, and tuberculosis [3]. Viral hepatitis is caused by at least five pathogenic hepatotropic viruses: hepatitis A (HAV), B (HBV), C (HCV), D (HDV), and E (HEV) viruses. For HAV and HEV, the fecal-oral route is the most predominant mode of transmission whereas HBV, HCV, and HDV are bloodborne viruses and are primarily transmitted through a breach in the skin (percutaneous) or mucosa (mucosal), invasive medical procedures using contaminated equipment and for HBV perinatal transmission and sexual contact. The discovery of HBV led to the first-ever vaccine not prepared by tissue culture but initially directly from plasma and soon the first vaccine produced by genetic engineering [4]. Hepatitis C virus (HCV) was the first virus that was identified by a direct molecular approach, without tissue culture, electron microscopy, or serology. Acute infections present with symptoms such as jaundice, dark urine, extreme fatigue, nausea, vomiting, and abdominal pain [5]. Epidemic jaundice due to hepatitis was first described by Hippocrates [5]. The hepatitis E virus (HEV) is the most important cause of epidemic hepatitis, whereas the hepatitis A virus (HAV) is more common among children [3]. Most acute liver failures diagnosed are attributable to HEV [6]. The National Viral Hepatitis Control Program was launched by the Ministry of Health and Family Welfare, Government of India, on 28th July 2018 on the occasion of World Hepatitis Day.

Dengue, complicated malaria, and typhoid have emerged as diseases affecting various organs during the period of active infection including the liver and brain. Hepatic involvement is usually subclinical but progresses to derangement of liver enzymes, increased bilirubin to clinical jaundice, and acute liver failure rarely [7]. In sepsis, the liver is injured by pathogens, toxins, or inflammatory mediators. Liver function tests (LFT) and disease-specific laboratory tests are helpful tools to diagnose different causes of hepatitis. Prevention is better than cure. Improvements in water quality and sanitation, screening of blood products before blood transfusion and antibody preparation as well as the inclusion of hepatitis A, and hepatitis B vaccine in childhood immunization programs have reduced the public health burden of hepatitis in India. The study's aim was to evaluate the clinical and serological profiles of different infections causing acute hepatitis in children admitted at a tertiary care centre as well as to describe the prevalence of different infections causing acute hepatitis in the pediatric age group in a hospital and to correlate the clinical and serological factors in infectious causes of acute hepatitis in children.

## Materials and methods

This was a cross-sectional study that was conducted over a period of 19 months from 1 September 2017 to 31 March 2019 in patients aged 1 to 18 years, admitted to our 120-bed multi-speciality and tertiary care centre, situated at Ring Road, Surat, in the Indian state of Gujarat. The sample size was calculated taking HAV in hepatitis cases as 76%, with a confidence interval of 10%, and taking design effect as 1. The sample size was initially 73 with an additional 10% in the case of a possible loss to follow-up during the study; the revised sample size was 80. Patients with clinically suspected hepatitis, laboratory evidence of infectious hepatitis, and a duration of illness of fewer than six months were included in the study. Patients with the non-infectious cause of hepatitis like drug-induced hepatitis, metabolic/genetic causes, and autoimmune hepatitis were excluded. The study was carried out after explaining the details to the parents or guardians by giving them the PIS (patient information sheet) and informed written consent was taken from guardians or parents on the informed consent form. Information and details of chief complaints, history of present illness, past history, family history, birth history, and previous drug history were taken by asking the parents or guardians. A thorough general examination and systemic examination were carried out. Reports of laboratory investigations and treatment profiles were entered in the proformas. The final diagnosis was made after necessary investigations like haemoglobin, platelet count, packed cell volume (PCV), white blood cell (WBC) count, peripheral smear (PS) for malaria, typhoid test, test for dengue, aminotransferase (AST) and alanine transaminase (ALT), total bilirubin, direct bilirubin, indirect bilirubin, anti-HAV IgM, anti-HEV IgM, urine for bile salts and bile pigments, blood culture and for hepatitis B HbsAg was done. The clinical and serological profile of the cases was presented in frequency and percentage for qualitative variables and in mean and standard deviation for quantitative variables. The prevalence of various causes was presented in percentage per 100 cases. The correlation of the clinical and serological factors with the causes of infective hepatitis in children was analyzed using bivariate analysis and statistical significance and was tested with the chi-square test for qualitative variables and t-test and ANOVA test for quantitative variables. Statistical significance was tested at a 95% confidence interval.

## Results

**Figure 1:**
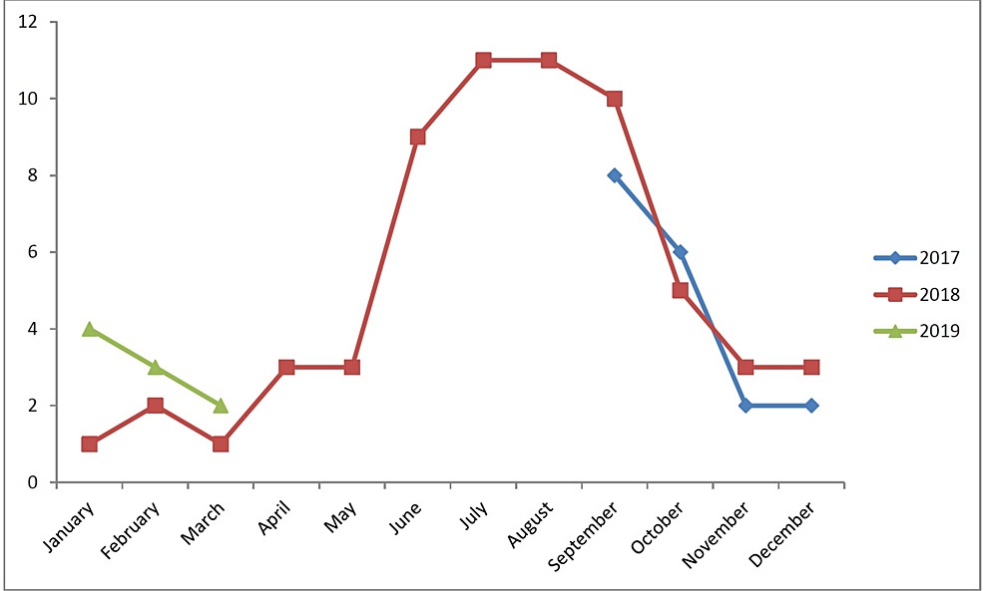
Distribution of study population as per year and month of presentation

This study was conducted over a period of 19 months from 1 September 2017 to 31 March 2019. There were 18 (20%) cases in 2017, 62 (70%) cases in 2018 and nine cases (10%) in 2019. Out of total of 89 cases, 60 cases (67%) were seen during the monsoon season; June (9), July (11), August (11), September (18) and October (11) (Figure 1).

In this study, 80% were males and 20% were females. The male versus female ratio was 4:1. Mean age was 8.8 years with a standard deviation of 4.4 years. The most common aetiology was viral hepatitis A (48.3%) followed by dengue fever and HAE. Rare etiologies comprised malaria, septicemia, and enteric fever. There was no case of hepatitis B and hepatitis C.

Patients with acute hepatitis had different complaints at the time of admission as depicted in Table 1. Common complaints were fever (90%), nausea (78.7%), dark yellow urine (68.5%), vomiting (56.2%), abdominal pain (41.6%), generalized weakness and body ache (39.3%), poor oral intake (33.7%), abdominal distension (10.1%), loose stools (6.7%), convulsion (3.4%), constipation (2.2%) and generalized swelling (1.1%). In both hepatitis A and E, patients had dark yellow urine (100%), nausea (100%), vomiting (100%), abdominal pain (50%), fever (50%), and poor oral intake (50%). In dengue, the majority of patients had fevers (90%), generalized weakness (75%), nausea (65%), abdominal pain (50%) and poor oral intake. In malaria, septicemia and enteric fever, common complaints were fever with chills and rigors, nausea, poor oral intake and generalized weakness.

**Table 1:** Relation between different etiologies of acute hepatitis and presenting complaints

Sr no.	Complaints	Hepatitis A	Hepatitis E	Hepatitis A & E	Dengue	Malaria	Enteric fever	Septicemia
1	Fever (80)	38 (88.4%)	10 (90.9%)	1 (50%)	18 (90%)	5 (100%)	4(100%)	4 (100%)
2.	Fever with chills and rigors (14)	3 (6.9%)	2 (18.2%)	0	4 (20%)	5 (100%)	0	0
3.	Nausea (70)	36 (83.7%)	10 (90.9%)	2 (100%)	13 (65%)	4 (80%)	4 (100%)	1 (25%)
4.	Dark Yellow urine (61)	43 (100%)	11 (100%)	2 (100%)	2 (10%)	1 (20%)	1 (25%)	1 (25%)
5.	Vomiting (50)	36 (83.7%)	10 (90.9%)	2 (100%)	1 (5%)	1 (20%)	0	0
6.	Abdominal pain (37)	21 (48.8%)	1 (9.1%)	1 (50%)	10 (50%)	1 (20%)	3 (75%)	0
7.	Generalized weakness. (36)	15 (34.9%)	1 (9.1%)	0	15 (75%)	3 (60%)	1 (25%)	1 (25%)
8.	Poor oral intake (30)	12 (27.9%)	8 (72.7%)	1 (50%)	5 (25%)	1 (20%)	1 (25%)	2 (50%)
9.	Abdominal distension (8)	8 (18.6%)	0	0	0	0	0	0
10.	Loose stools (6)	4 (9.3%)	0	0	2 (10%)	0	0	0

On systemic examination, common signs found were hepatomegaly (60.7%), abdominal tenderness (58.4%), splenomegaly (10.1%) and ascites (11.2%). During the general examination, 11.2% were found to have bleeding manifestations, 7.9% had hypotension, 1.1% had hypothermia, and 4.5% had restlessness. In combined viral hepatitis A and E, patients had hepatomegaly (100%), icterus (50%) and abdominal tenderness (100%) (Table 2).

**Table 2:** Relation between different etiologies of acute hepatitis and clinical findings

Sr no.	Clinical finding	Hepatitis A	Hepatitis E	Hepatitis A & E	Dengue	Malaria	Enteric fever	Septicemia
1	Icterus (62)	38 (88.4%)	11 (100%)	2 (50%)	8 (40%)	1 (20%)	1 (25%)	1 (25%)
2	Hepatomegaly (54)	29 (67.4%)	4 (36.4%)	2 (100%)	12 (60%)	3 (60%)	2 (50%)	2 (50%)
3	Abdominal tenderness (52)	37 (86%)	8 (72.7%)	2 (100%)	4 (20%)	1 (20%)	0	0
5	Bleeding manifestations (10)	1 (2.3%)	0	0	5 (25%)	3 (60%)	0	1 (25%)
6	Ascites (10)	4 (9.3%)	1 (9.1%)	0	5 (25%)	0	0	0
7	Splenomegaly (9)	7 (16.3%)	0	0	1 (5%)	0	1 (25%)	0
8	Hypothermia (1)	0	0	0	0	0	0	1 (25%)
9	Drowsiness (4)	0	0	0	2 (10%)	0	0	2 (50%)
10	Restlessness (4)	2 (4.6%)	0	0	2 (10%)	0	0	0

In laboratory analysis, the majority (73%) of patients had normal WBC count whereas 20.2% of patients had increased WBC count and 6.7% of patients had decreased WBC count. Around 80% of patients were found to have normal platelet count, whereas 20% of patients had thrombocytopenia. Patients with thrombocytopenia were classified into three categories: 7.9% of patients had mild thrombocytopenia, 5.6% had moderate thrombocytopenia and 6.7% had severe thrombocytopenia. Around 65.2% of total cases (89) were found to have bile salts and bile pigments on urine examination.

Among all patients, 3.4% tested positive for Plasmodium vivax and 2.2% were positive for Plasmodium falciparum malaria. Blood culture was done for eight patients. Among them, 50% of the patients had Salmonella typhi growth. In the remaining four patients no growth of any organism was detected.

All patients in this study had AST and ALT values of more than 100 IU/L. The majority of the patients (57.3%) had ALT between 100 to 1000 IU/L, 38.2% of patients had ALT between 1000 to 5000 IU/L and the remaining 4.5% had an extreme rise in ALT value of more than 5000 IU/L.

As evident from Table 3, 100% of cases of hepatitis A and hepatitis E were diagnosed by the presence of IgM antibodies in serum against the respective virus. In dengue, 70% of cases had dengue NS1 antigen test positive whereas 15% of cases had dengue IgM and 15% of cases had dengue IgG antibody test positive. Out of all cases of enteric fever (4), only one case was found to be IgM positive on typhidot whereas blood culture was positive (growth of Salmonella typhi) in 100% of cases.

**Table 3:** Distribution of study population as per diagnostic tests

Sr no.	Diseases	Serological test	Frequency	Percentage
1	Hepatitis A	Anti Hepatitis A virus IgM antibody	43	100%
2	Hepatitis E	Anti Hepatitis E virus IgM antibody	11	100%
3	Dengue	Dengue NS1Ag	14	70%
		Dengue IgM	3	15%
		Dengue IgG	3	15%
4	Enteric fever	Typhi dot test	1	25%
		Blood Culture	4	100%

In this study, out of the total cases (89), 4.5% of cases had complications; the most common complication was hepatic encephalopathy which was seen in patients of hepatitis A, dengue and septicemia (2.3%, 5% and 25% respectively). Another complication was septic shock with multiorgan dysfunction in the case of septicemia. The hospital stay was less than five days in 88% of patients, 5-10 days in 8% and more than 10 days in 4.5% of patients. Among all 89 cases, 98.9% cases recovered well and were discharged. One patient died due to septicemia with multi-organ dysfunction.

## Discussion

The causes of acute hepatitis vary with the geographical location and it depends upon the prevalent infectious agents. In this study, the commonest etiologies for acute hepatitis in children were viral hepatitis A (48.3%), dengue fever (22.5%) and viral hepatitis E (12.4%). Rare etiologies were malaria (5.6%), septicemia (4.5%), enteric fever (4.5%) and combined viral hepatitis A and E infection (2.2%) As compared to other studies, no case of hepatitis B and C was found in this study. The reason for this may be a small sample size and different studies conducted in different geographic locations in different setups.

In this study, the majority of cases (67%) were seen in monsoon months (July to October). Singh J et al. study also found that 59% of cases of viral hepatitis were seen in the months from June to September [8]. Fares’ study on the seasonality of hepatitis found no definite and consistent seasonal pattern observed in viral hepatitis [9]. Findings indicate that HAV infections are transmitted indirectly through rainfall. Therefore, disruption of sanitation and water supplies results in the seasonal occurrence of hepatitis A and E.

In this study, 25.8% of cases were in the preschool age group, 33.7% of cases were in school going age group and 40.5% cases were adolescents. A higher seroprevalence of HAV was observed among the older children than among the younger children. Girish et al. found that the adolescent age group was affected more as 80% of cases were more than 10 years of age [5]. A study by Mathur et al. found that with improvements in the economic and living conditions of the communities, the age of acquiring hepatitis A virus (HAV) infection is shifting from early childhood to adolescence and young adulthood [10]. This may be due to a change in their food habits like eating unhygienic food, resulting in changing epidemiology. In this study, the hospital stay was less than 10 days for 95.5% of patients, whereas it was more than 10 days for 4.5% of patients.

Among patients who had fevers, 15.7% had fevers with chills and rigors. In this study, we found that dengue cases had a more generalized weakness as compared to other patients. Fever with chills and rigors was found more in cases of malaria. Girish et al. [5] in their study found that fever was the presenting symptom in all cases (100%), jaundice and vomiting in 83.3% of cases each, followed by dark-coloured urine (70.8%), abdominal pain (68.7%), nausea (16.7%), anorexia, loose stools each accounting 8.3% and altered sensorium in 6.2%. Salahuddin et al. [11] in their study on the spectrum of acute viral hepatitis in children found that most of the children presented with jaundice (100%), anorexia (100%), nausea and vomiting (88%), low-grade fever (65%), with right upper abdominal pain (73%); therefore, in acute hepatitis, clinical presentations can vary. In this study, on general and systemic examination, the most common clinical finding was icterus (69.7%). Other common signs were hepatomegaly (60.7%), abdominal tenderness (58.4%), splenomegaly (10.1%) and ascites (11.2%). On general examination, 31.5% had anaemia, 11.2% had bleeding manifestations, and 7.9% had hypotension. Bleeding manifestations were mainly present in cases of dengue, malaria and septicemia. Icterus was present mainly in hepatitis A and hepatitis E infections. Other signs were similar in all causes. In their study, Girish et al. [5] found that 100% of cases had icterus, 76% cases had hepatomegaly, 35.4% had pallor, 20.8% had edema, 16.7% had ascites and 10.4% had splenomegaly [5]. Hence, the study by Girish et al. and the present study found similar signs in acute hepatitis cases, except in the present study, 69.7% had icterus and 11.2% had bleeding manifestations whereas, in the study by Girish et al., all patients had icterus and no bleeding manifestation was found in any case. Soni et al. [12] in their study found that out of the total patients with hepatitis, 72.5% had icterus. In the present study and the study by Soni et al., icterus was present in almost 70% of cases of hepatitis, hence, the sensitivity of icterus is considered as 70% for the diagnosis of hepatitis [12]. Therefore the absence of icterus does not rule out hepatitis. Thus we cannot differentiate etiologies based on clinical findings alone. In this study, 20% of patients had thrombocytopenia with a platelet count of less than 1,50,000/µl. Mean platelet count was decreased in dengue fever and malaria. Kotepui et al. [13] in their study found that patients with platelet counts < 150,000/ul were 31.8 times (odds ratio) more likely to have a malaria infection. Thrombocytopenia was present in 84.9% of malaria-infected patients and was independent of age, gender and nationality (P value < 0.0001). In this study, thrombocytopenia was present in 60% of malaria cases.

Pothapregada et al. [14] in their study found that the haematological parameters showed anaemia (29.5%), leukopenia (19.1%) and thrombocytopenia in 82.4% cases of dengue fever. 

In this study, all patients had ALT and AST values of more than 100 IU/L. Maximum patients (57.3%) had ALT between 100 to 1000 IU/L, 38.2% of patients had ALT between 1000 to 5000 IU/L and the remaining 4.5% had a value of more than 5000 IU/L. Girish et al. [5] in the study found that 56.3% of cases had ALT between 100 to 1000 IU/L and 43.7% of cases between 1000 to 5000IU/L. The findings were similar to the present study.

Most of the patients (93.7%) in the present study had total bilirubin levels between 1 to 10 mg/dl, and 5.6% of patients had total bilirubin of more than 10 mg/dl. These findings are similar to the Girish et al. [5] study in which 87.5% of cases had serum bilirubin between 1 and 10 mg/dl and 12.5% had bilirubin more than 10 mg/dl. In this study, 100% of cases of both hepatitis A and hepatitis E were diagnosed by positive anti-HAV and anti-HEV IgM antibody tests respectively. No HBsAg-positive case was found in our study. Similarly, Girish et al. [5] found all cases of hepatitis A and E had antibody tests positive and 2.08% of cases had HBsAg positive.

In this study, among all cases of dengue, 70% of cases were found to have dengue NS1 antigen positive whereas 15% of cases had dengue IgM and 15% cases had dengue IgG positive. Out of the total cases of dengue fever (20), 60% of cases had dengue without warning signs, 35% of cases had dengue with warning signs and 5% cases had severe dengue. In all three classes, the majority of cases were diagnosed by the dengue NS1Ag test (70%, 66.7% and 100% cases respectively). In dengue, after the onset of illness, the virus can be detected in serum, plasma, circulating blood cells and other tissues for four to five days. During the early stages of the disease, virus isolation, nucleic acid or antigen detection can be used to diagnose the infection. At the end of the acute phase of infection, serology is the method of choice for diagnosis [15].

In this study, among the total cases of enteric fever (4), one case had typhidot test positive. All cases showed Salmonella typhi growth on blood culture. So, out of four cases with positive blood culture, one case had a positive typhidot test. Jesudason et al. [16] in their study observed that the typhidot test gave a sensitivity of 100 % and specificity of 80% when bacteremic patients were analysed and study by Krishna et al. [17] observed that the sensitivity, specificity, positive and negative predictive values of typhidot test using blood culture as gold standard were 100%, 95.5%, 89.2%, and 100%, respectively for typhoid fever. So there is a need for proper evaluation between the two tests in studies with an increased number of enteric fever cases.

In this study, out of the total cases (89), complications were present in 4.5% of cases whereas Girish et al. [5] found complications such as hepatic encephalopathy in 8.3% of cases. Similarly, in this study, the most common complication was also hepatic encephalopathy seen in patients of hepatitis A, dengue and septicemia (2.3%, 5% and 25% respectively). Another complication observed was septic shock with multi-organ dysfunction in one case of septicemia.

In this study, the majority of cases had viral hepatitis and dengue fever. Hepatitis (A and E) and dengue fever have no specific treatment available at present. Recovery from symptoms may be slow and take several weeks or months. Therapy is aimed at avoidance of unnecessary medications, maintaining comfort, adequate nutritional balance, and prevention of complications. So, most of the patients were discharged within two to three days. Some cases with complications like septicemia with septic shock, and dengue with hepatic encephalopathy would require longer hospitalization. 

## Conclusions

The most common cause of infective hepatitis in children is hepatitis A. Other causes like dengue, malaria and typhoid should also be kept in mind. Clinical presentation in acute infective hepatitis is non-specific and variable. Due to variable clinical presentations, lab diagnosis including serology becomes important to further manage a case of hepatitis. The absence of icterus does not rule out hepatitis. As prevention is better than cure, the spread of hepatitis A and E can be reduced by adequate supplies of safe drinking water, proper disposal of sewage within communities and personal hygiene practices such as hand-washing with soap and water. Timely immunization against hepatitis is strongly recommended. It is also important to educate the society regarding clinical presentation, and different etiologies of acute infective hepatitis of disease so that they can seek medical intervention early and prevent complications and mortality associated with the disease.
